# An Interoperability Framework for electromobility (INFRA): The main results from the USER-CHI framework implementation in a new spotlight

**DOI:** 10.12688/openreseurope.14510.1

**Published:** 2022-05-25

**Authors:** Katharina Csillak, Mariana Moreno Kuhnke

**Affiliations:** 1 Mobility Department, Institute for Climate Protection, Energy and Mobility (IKEM), 10179 Berlin, Germany; 2 Energy Law Department, Institute for Climate Protection, Energy and Mobility (IKEM), 10179 Berlin, Germany

**Keywords:** Electromobility, charging infrastructure, interoperability, electric vehicle, framework, USER-CHI, INFRA, legal requirements

## Abstract

In 2021 the number of electric vehicles (EV) circulating in the European Union (EU) was higher than ever before. The innovative solutions of the user centric charging infrastructure (USER-CHI) project aim at devising the basic guidelines for an interoperable charging infrastructure for EV. The future goal is for users to be able to charge “anywhere, anytime”. USER-CHI works towards a large-scale e-mobility market and the development of integrated smart solutions and new business models. These will be demonstrated in five urban areas, which are part of the Trans-European Transport Network corridor (Barcelona metropolitan area, Rome, Berlin, Budapest, and Turku).

To make access to the charging infrastructure possible irrespectively of vehicle brands and operators, an analysis of the existing interoperability framework was considered essential within the USER-CHI project. Pursuant to this, the Institute for Climate Protection, Energy and Mobility (IKEM) developed INFRA (Interoperability Framework) **,** a study that contains an analysis of the four layers that condition interoperability in the electromobility sector (organizational, semantic, technical and legal). Building on this analysis, the existing barriers for interoperability in each layer were identified and guidelines and recommendations were elaborated. The baseline of this paper are the role schemes, guidelines and recommendations elaborated for INFRA summarized in minimum requirements within each layer. The INFRA results are already available on CORDIS.

In addition, further desk research shows the latest developments in the EU regarding electromobility and puts INFRA in the context of the most recent discourse. The new insights of this paper offer an updated overall short summary of the main requirements for an interoperability framework for electromobility in the EU.

In conclusion the current developments underline the importance of the minimum requirements identified in INFRA and first steps towards a more interoperable infrastructure system have been taken.

## Plain language summary

In 2021 the number of electric vehicles in the European Union (EU) was higher than ever before. Therefore, the charging infrastructure in Europe has many challenges. The EU project USER-CHI (user centric charging infrastructure) aims at devising the basic guidelines for an interoperable charging infrastructure in INFRA (Interoperability Framework). The goal is, to charge "everywhere, anytime". The first part of the paper is about the main results from the framework. The second part presents additional desk research. This allowed for the identification of new aspects that were not, or only superficially, considered during INFRA. As result, the most important aspect to achieve interoperability in the EU is a user friendly charging infrastructure. It needs to fill the gaps in the European legislation and transfer them into national legal frameworks, otherwise it causes wide disparities between countries. Nevertheless, the further success of the massive uptake of electric vehicles depends on solutions that fit the needs of electric vehicle users.

### Glossary

**Table:** 

**ACEA**	The European Automobile Manufacturers’ Association
**AFID**	Alternative Fuels Infrastructure Directive
**AFIR**	Alternative Fuels Infrastructure Regulation
**APDS**	Alliance for Parking Data Standards
**AVERE**	The European Association for Electromobility
**B2B**	Business to Business
**CLICK**	Charging Location and Holistic Planning Kit
**CPOs**	Charging Point Operators
**DSO**	Distribution System Operator
**eMIP**	Electro Mobility Inter-roaming Protocol
**EEMEC**	European E-Mobility Expertise Center
**EMEurope**	Electric Mobility Europe
**EMSPs**	Electromobility Service Providers
**EU**	European Union
**EV**	Electric Vehicle
**EVSE**	Electric Vehicle Supply Equipment
**GDPR**	General Data Protection Regulation
**IDZ**	Initiative Deutscher Zahlungssysteme *(Initiative of German payment systems)*
**IKEM**	Institute for Climate Protection, Energy and Mobility
**INCAR**	Interoperability, Charging and Parking Platform
**INFRA**	Interoperability Framework
**LEV**	Light Electric Vehicles
**MID**	Measuring Instruments Directive
**OCHP**	Open Clearing House Protocol
**OCPP**	Open Charge Point Protocol
**OICP**	Open InterCharge Protocol
**P&C**	Park and Charge
**TCR**	Technical Connection Rules
**TEN-T**	Trans-European Transport Network
**USER-CHI**	Innovative solutions for USER centric CHarging Infrastructure
**V2G**	Vehicle to Grid
**WEVCS**	Wireless Electric Vehicle Charging Systems

## 1 Introduction

In the year 2020 the global electric passenger car stock almost reached the 10 million electric cars. In 2020 Europe had the second highest electric vehicle (EV) stock in the world with 3.3 million electric vehicles, after China (total: 5.4 million) and before the United States (total: 1.8 million) ^1^. Thereof, 1.4 million Plug-in-Hybrid Electric Vehicles (PHEV) and 1.8 million Battery Electric Vehicles (BEV) were part of the European EV stock ^2^. The significant growing sales share of new electric cars in Europe (that raised from 0.5% in 2013 to 10.5% in 2020) shows an increasing number of EV users ^3^. This growth requires unified standards for the charging infrastructure across the European Union (EU) that allows the rollout of an interoperable publicly accessible charging infrastructure along the Trans-European Transport Network (TEN-T) corridors with charging options at home and at work. The aim is to increase the convenience of the charging possibilities for electric vehicle owners ^4^.

The Horizon-2020 project USER-CHI ^5^ targets the development of guidelines for a user-friendly charging infrastructure. The goal is that every EV driver has the possibility to access an interoperable charging infrastructure “anywhere” and “anytime” across the EU. USER-CHI supports the mentioned large-scale e-mobility market roll out with the development of smart solutions and business models while working on the necessary framework conditions. The five demonstration cities are connected through the key Mediterranean and Scandinavian TEN-T corridors: Rome (Italy), Berlin (Germany), Turku (Finland), Barcelona metropolitan area (Spain) and Budapest (Hungary). Additionally, the cities Murcia (Spain) and Florence (Italy) are part of the project as replication cities, covering in this way several different city typologies. That means that the results of the project will have a higher impact, and gives the possible solutions for user-friendly e-mobility a higher replicability in a greater number of cities in Europe.

Within the USER-CHI project and for the elaboration of the Deliverable D3.2. Interoperability Framework (INFRA) ^6^, IKEM (Institute for Climate Protection, Energy and Mobility) analysed the existing interoperability framework to identify the minimum requirements for a unified and user-friendly roll out of the EV charging infrastructure across Europe in tight cooperation with the project partners like demonstration cities and product developers. To this end, the main existing barriers that currently hinder the aimed user-friendly roll out of this infrastructure were identified and, to overcome them, guidelines and recommendations were developed. INFRA was delivered in May 2021 ^7^.

This paper aims at giving publicity to the results of INFRA and adds some new perspectives. In this sense, it is based on the results of INFRA and further desk research on the identified minimum requirements. The INFRA deliverable is the main source of information for the elaboration of this publication. The main results of INFRA (role schemes, guidelines and recommendations) are summarized here in the form of minimum requirements for the interoperability of the EV charging infrastructure across the EU. This means that additional perspectives and information have been added to the results of INFRA by the authors.

## 2 Methods

This paper was elaborated based on the results of the deliverable D3.2. Interoperability Framework (INFRA) ^8^ of the USER-CHI project (see *Data availability*). Thus, first the results of INFRA were summarized in minimum requirements. Additionally, new findings were made as a result of the desk research and added to this publication. The steps taken are described in detail in the following.

### 2.1 INFRA results

The role schemes, guidelines and recommendations described in INFRA are summarised in this paper as minimum requirements ^9^ to achieve the roll out of an interoperable charging infrastructure across the EU. These requirements constitute the baseline for the development of this paper.

This paper is structured in a continuous text, in an attempt to make it more reader friendly. The authors have transparently identified the authorship through citations and footnotes. Most citations from other literature are linked to the desk research (if they are not, a further footnote will identify the paragraph as a result from the input of the INFRA deliverable).

The input from the INFRA deliverable in each of the minimum requirements is clearly identified through a footnote, that either states that it is information contained in INFRA or directly citates the deliverable “Nowack *et al* (2021)”. The authors own input resulting from the desk research is also clearly identified as such through a footnote.

The INFRA deliverable was conceived as a product of USER-CHI and offers a first overview of an interoperability framework for electromobility. As for the methodology, the INFRA deliverable was developed as explained below.

The layers that condition interoperability were identified. The layers identified as mainly relevant were the organisational layer (global layer), the technical, semantic, and legal layer (specific layers). Interdependencies between all layers were identified during the work for the INFRA deliverable. The organizational layer, also called global layer, gives an overview of all stakeholders involved in the roll out of the charging infrastructure and achieving interoperability. The technical and the semantic layer focus on technical requirements for interoperability (like soft- and hardware). And last, the legal layer reflects the (inter-)national legal situation that conditions the interoperability of the EV charging infrastructure.

The INFRA deliverable was developed in an interactive process: a) a validation workshop ^10^ was held in each demonstration city with participation of the product developers and city partners to develop stakeholders (global layer) and b) an inventory of technical (semantic and technical) and legal requirements was developed based on the basis of the input of the deliverable D1.3 “Technical and Legal Requirements” of the USER-CHI project (specific layer). In step c) these requirements were clustered according to the priorities of the demonstration cities. In the last step d) the results were validated by each demonstration city via workshops ^11, 12^.

As a result, the INFRA deliverable identified and categorized the role schemes, guidelines and recommendations to achieve the interoperability of a cross borders charging infrastructure for EV ^13^: a selected set of technical, semantic, and legal guidelines determining interoperability were consolidated, the main barriers to achieve interoperability in relation to the identified specific aspects within each layer were identified and recommendations on how to diminish the main barriers were developed.

### 2.2 Desk research

For this paper, further research has been carried out on the scientific literature regarding the requirements identified in the INFRA deliverable as essential to achieve the roll out of an interoperable charging infrastructure for EV across the EU. In this sense, the most important aspects of the identified minimum requirements have been in this paper further developed as a result of a wide literature review.

As already mentioned in Section 2.1, the information from INFRA on which this paper is based, is either identified with the citation of the deliverable “Nowack *et al* (2021)” in footnotes or the footnote includes a reference to the INFRA deliverable.

The literature review focused on research papers and statements of researchers, non-profit-organisation, networks and clusters of the electromobility field between June 2021 and December 2021 (cut-off date: December 15 ^th^, 2021). The literature was sourced by key words of the selected minimum requirements of INFRA with a “snowball sampling technique”. The literature that was found was then used to find similar or familiar literature, that is connected to it (through the bibliography, recommended publications, or references and citations). The technique is classified as part of the non-systematic literature studies and has been conducted manually. However, the “snowball sampling method” is a common technique in qualitative research ^14^, as well as in systematic literature review processes ^15^. In 2020 “up to 51% of references in a systematic review are identified by *snowballing*” ^16^. In conclusion, the results of the literature review complement the summarised minimum requirements (cited as Nowack *et al.* (2021)) with information and current updates that were found via ‘ *snowballing*’. The authors own input resulting from the desk research is clearly identified as such through a footnote.

In this sense, an update of the developments achieved (semantic and technical) since the drafting of the INFRA deliverable has been made and the legal status quo of each of the legal minimum requirements has been revised. Therefore, the results of the desk research reflect the most recent developments. This desk research allowed us to identify new aspects that were not – or only superficially – considered during the elaboration of the INFRA deliverable. In this sense, the desk research gives new inputs to the results of the INFRA deliverable, giving them a wider contextualisation in the scientific discourse regarding interoperability in the European electromobility sector.

## 3 Results: Minimum requirements for the roll out of an interoperable EV charging infrastructure across the EU

**Figure 1.**
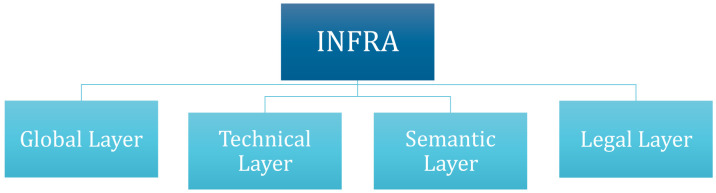
Overview of INFRA (Interoperability Framework) layers. Own presentation.

As a consequence of the EU CO _2_ emission reduction targets ^17^, the number of EVs in the EU will increase considerably in the coming years. This presents a challenge for the EV charging infrastructure and for the national and European electric grids. To make sure that the charging infrastructure is interoperable across the EU and that the electric system can withstand such an increase in electricity consumption, a set of minimum requirements was identified in the INFRA deliverable and categorised in the organizational layer (global layer) an in the technical, semantic and legal layer (specific layers). An overview of the layers within the INFRA deliverable is shown in Figure 1.

Following this, a short introduction on the importance of each specific layer for the cross borders interoperability of the EV charging infrastructure is made. Furthermore, the identified minimum requirements within each layer are displayed together with an explanation of their role in achieving interoperability, possible barriers that hinder their complete fulfilment and, if existing, concrete solutions to overcome these barriers are proposed. Baseline for the development of the minimum requirements are the results of INFRA. However, these have been updated, and some new requirements have been added and merged with them.

As already mentioned, all layers are interconnected. This means that, while some technical and semantical essential aspects to the interoperability of the charging infrastructure are identified and described in the respective layer, they are also mentioned in the legal layer because they need to be foreseen in the legal instruments at EU level in order to achieve a charging infrastructure with unified characteristics in all EU member states. Thus, they are also briefly mentioned in the legal layer.

### 3.1 Minimum requirements within the global layer

The organisational layer creates the context and sets the basic structures and conditions for the semantic, technical, and legal layer. To achieve the roll out of an interoperable EV charging infrastructure across the EU, all stakeholders that have a role in the intended practical implementation of the interoperable charging infrastructure need to achieve a consensus on the pursued goals, the distribution of roles and the assumption of responsibilities ^18^. In this sense, all essential roles must be clearly identified and assumed by a concrete stakeholder.

Essential to achieve interoperability within the global layer is, in any case, (a) the involvement of each stakeholder in the organization and execution processes at an early stage to avoid later changes, and (b) a barrierless and fluid communication and cooperation between stakeholders. The INFRA deliverable showed that essentially the same stakeholders appeared in all demonstration cities, 21 roles were found but not every role was represented in every city, and some stakeholders had more than one role ^19, 20^.

**Figure 2.**
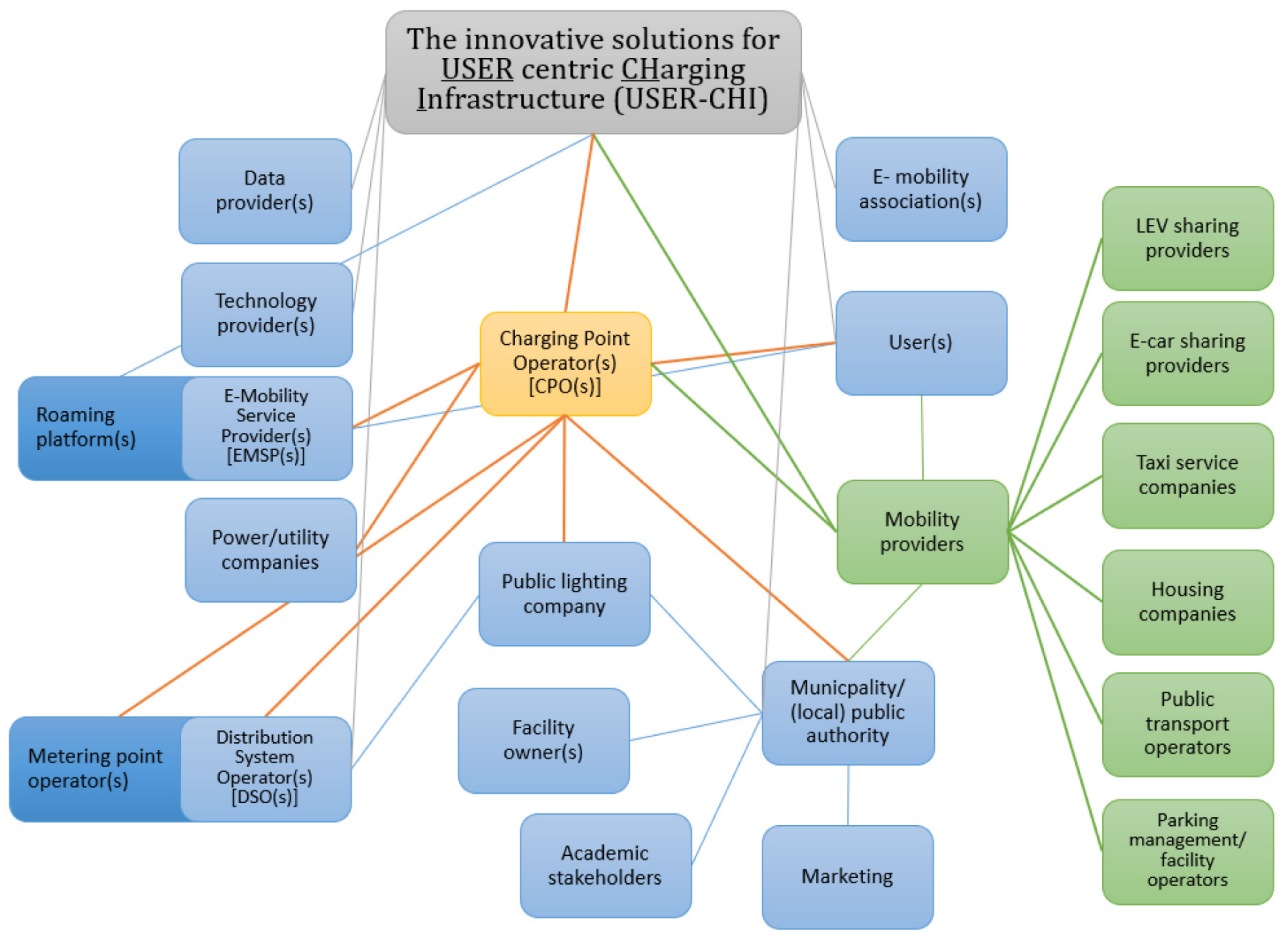
Overall role scheme of the e-mobility ecosystem in user centric charging infrastructure (USER-CHI). LEV, Light Electric Vehicles. Figure 2 has been reproduced with permission from Nowack *et al.* (2021), p. 21.

The results of the organisational layer of INFRA reflect very accurately the status quo in the scientific literature on this matter ^21^. Thus, the importance of clear roles in an interoperable electromobility infrastructure is mentioned in other Horizon 2020 projects like NeMo ^22^. The consolidated models of different actor groups like IT-suppliers, decision makers and public authorities and their relationship with each other show the same results as INFRA (see Figure 2). For example, the needs and interactions of the involved stakeholders to achieve interoperability and cooperation within the European Electromobility Network have already been summarised ^23^. This network was the starting point of the creation of an eRoaming hyper-network within their project, where actors will be able to interact and exchange data ^24^. In addition, clear role schemes of all actors and their assignments have an important impact and the partnership´s dynamic is an element, that needs to be extended and institutionalized ^25^. In conclusion, the involvement of each stakeholder in the implementation process (a) could be a challenge, when there is a high dynamic, but also, (b) could be successful, if it is integrated early in the process of creating a network. External organisations or networks like e.g., AVERE (The European Association for Electromobility) ^26^, EMEurope (Electric Mobility Europe) ^27^ or EEMEC (European E-Mobility Expertise Center) ^28^ could be helpful to connect and exchange experiences with other project consortiums. Additionally, International Energy Agency (IEA) ^29^ and European Alternative Fuels Observatory (EAFO) ^30^ offer further information and networking possibilities. They also offer information on summarized statistic and data about electromobility. In any case, communication between stakeholders is a key element to achieve the interoperability of the EV charging infrastructure.

### 3.2 Minimum requirements within the specific layers

The specific layers (the technical, the semantic and the legal) contain the aspects that condition interoperability in a practical way. Therefore, the readiness status of science and politics to achieve the interoperability of the EV charging infrastructure was analysed in INFRA. For this, the guidelines and recommendations elaborated in the INFRA deliverable have been summarised and used as baseline for this paper in form of minimum requirements. Additionally, new insights on the developments within each layer in relation to different aspects have been added to the respective requirements. The information from the INFRA deliverable and the information from the desk research have been therefore merged. Nevertheless, every paragraph has a footnote that indicates weather the input comes from the INFRA deliverable or it is the author´s own input. Information from the INFRA deliverable is either cited as “Nowack *et al.* (2021)” or marked as such in a footnote.

**Figure 3.**
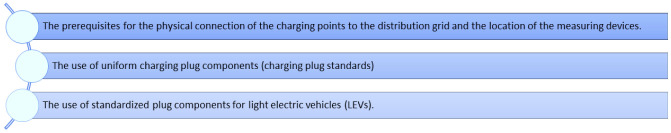
Summarised minimum requirements of the technical layer on basis of INFRA (Interoperability Framework) input. Own presentation.

***3.2.1 Technical layer.*** Within the technical layer, the literature about the essential technological conditions (hardware) to achieve the interoperability of the charging infrastructure of EV across the EU was analysed. In this sense, the results from the INFRA deliverable were summarized (as mentioned in Section 2) in three “minimum requirements” ^31^ (see Figure 3). Additionally, some further desk research on the development and status of these technologies and further technological applications that condition interoperability has been conducted. The results of INFRA have been systematised as follows and have been complemented with information resulting from the desk research (for further information on the authorship see citations and footnotes).

The first minimum requirement is related to the energy supply and the metering devices of the charging points. In this sense, the prerequisites for the physical connection of the charging points to the distribution grid and the requisites on the location of the measuring devices established by local grid and metering point operators must be observed. There are standards and Technical Connection Rules (TCR) for the connection to the grid and rules for the measuring device’s location on regional, national, and international level that need to be complied. All these issues, together with potentially necessary grid extensions should be identified at the early stages of the planning process ^32^.

The use of uniform charging plug components ^33^ (charging plugs standards) is the second minimum requirement for interoperability in the electromobility sector within this layer. There already are standards in place for these plugs at international and at European level. In this sense, the use of “ *Type 2 sockets or cables for AC charging points and Combo 2 sockets or cables for DC charging points*” ^34^ is already a requirement at European level and was established through Annex II of the Alternative Fuels Infrastructure Directive (AFID) ^35^. For more information about the AFID see section 4.2.3 dealing with the legal layer ^36^.

The third minimum requirement deals with plug components for Light Electric Vehicles (LEV) ^37^ charging ^38^. Thus, the use of standardized plug components is of high importance. However, unified standards have not been established yet at every level and each provider uses its own plug components.

There are plenty of other charging solutions next to self-reliant solar-powered battery charging scheme for LEVs ^39^ or onboard charger (OBC) ^40^. Plenty of experiences have already been made on the difficulties that the lack of initial standards causes for the acceptance and access of new EV to the charging infrastructure. Thereafter, special and concrete requirements for LEVs must be quickly integrated into EU legislation. An example is the introduction of small three– or four-wheeled electric vehicles (SEVs) ^41^, that is currently being discussed and is, again, leading to new technical challenges for the EV charging infrastructure ^42^.

The same applies to wireless charging. Several different technologies for wireless charging already exist ^43^ and this kind of EV charging will probably incur a complex situation in the next years since there are no requirements established for the technical components for wireless charging yet (technology is still in development). These Wireless Electric Vehicle Charging Systems (WEVCS) are currently rare in most countries and the discussion is mostly based on the techno-economic aspects ^44^. This fact has the risk that this technology remains as a “niche product” ^45^ because it is not interoperable. However, it could foster the discussions about smart road concepts ^46^ and more hard- and software applications on street.

The fourth minimum requirement consists of the implementation of a smart metering infrastructure ^47^, which’s importance is already mentioned in the AFID ^48^. Smart meters are considered as an improvement of the public charging points that allows to monitor the energy flowing process and gives the user the possibility start and stop the charging process in order to save energy ^49^. Among other things, smart meters could track and submit the charging information to the supplier to ensure accurate bills. However, for smart meters to be able to operate accurately the interoperability of this technology needs to be guaranteed across the EU.

In conclusion, the technical possibilities are growing rapidly and technological developments occur on a daily basis. Therefore, the technological development in matters of interoperability needs to be closely followed. On the other hand, the adoption of minimum universal requirements for components of EV at early stages is very important to ensure interoperability within the technical layer.

**Figure 4.**
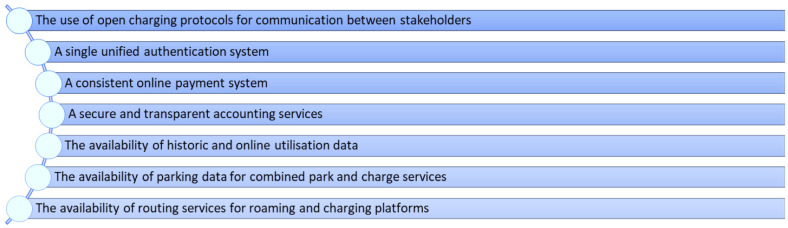
Summarised minimum requirements of the semantic layer on basis of INFRA (Interoperability Framework) input. Own presentation.

***3.2.2 Semantic layer.*** The semantic layer contains the communication aspects between service providers (stakeholders). Achieving semantic interoperability means providing “ *a common meaning of the data exchanged by heterogeneous devices, even if they belong to different domains*” ^50^. In this sense, seven minimum requirements ^51^ for syntactic and communication that condition the interoperability of the charging infrastructure for EV have been summarised within the semantic layer (see Figure 4) based on the input of the INFRA. Further desk research was conducted on these minimum requirements. The minimum requirements have been systematised as follows merging the input of the INFRA deliverable and the results of the desk research (for further information on the authorship see footnotes and citations).

The first minimum requirement within this layer is the use of open charging protocols for communication between stakeholders, such as OICP (Open InterCharge Protocol) ^52^ for roaming and authentication or OCPP (Open Charge Point Protocol) ^53^ for communication between Electric Vehicle Supply Equipment (EVSE) and Charging Point Operators, (CPOs). Currently, semantic communication and data exchange are not uniformly guaranteed because stakeholders use different proprietary protocols (OICP, Open InterCharge Protocol; OCHP, Open Clearing House Protocol; eMIP, Electro Mobility Inter-roaming Protocol) or customised interfaces ^54^.

The use of a single unified authentication system or the possibility to authenticate at public charging stations through any of the available systems on the market ^55^ is the second minimum requirement. Currently, every CPO uses a different authentication system and EV drivers are forced to have several different ways to authenticate to be able charge their EVs at different charging points. Essential for interoperability is also the existence of consistent/uniform online payment systems ^56^ between stakeholders and users that consider the complexity of roaming services, being this the third minimum requirement. For general and specific applications, different providers of online payment services are available. This situation does not foster interoperability. Therefore, the utilization of an online payment system that doesn´t depend on any operator could, in this case, be a good solution. Linked to this is the use of secure and transparent accounting services ^57^ (fourth minimum requirement) ^58^. Additionally, the application of the blockchain technology could contribute to achieve interoperable and secure accounting processes amongst stakeholders ^59^.

Steps towards the achievement of unified payment systems that fosters an interoperable EV charging infrastructure have already been taken. An example of this is the change introduced in the “Charging station ordinance” (‘ *Ladesäulenverordnung*’) ^60^ in Germany. This ordinance foresees that, from July 2023 on, CPOs are obliged to offer users the possibility to pay with credit card. Currently the option to pay with EC/credit card is the most attractive option for EV users and a good known alternative to this is ‘PayPal’, as a digital payment service provider ^61^. Countries like Greece, Poland, France, and Slovenia have the highest rates of payment with credit card ^62^. In conclusion, a unified payment system with credit card across the EU charging infrastructure would clearly foster the interoperability of the EV charging infrastructure ^63^.

As already mentioned, the ordinance applies only to the German charging infrastructure, but the EU also plans to simplify the payment system by introducing modifications to the AFID ^64^ with the proposal for the new Alternative Fuels Infrastructure Regulation (AFIR) ^65^ to make the payment service “ *as simple as possible for consumers*” ^66^. In this sense, there are several stakeholders ^67^, made comments to the AFIR proposal and many of them recommend the introduction of the obligation to allow credit card payments ^68^ at public charging points as an essential requirement to achieve the interoperability of the charging infrastructure across the EU ^69^.

Back to the requirements ^70^: to achieve a well-planned roll out of the EV charging infrastructure, the availability of historic and online utilisation data ^71^, in compliance with the data protection regulations, is necessary for administrations to be able to coherently plan the expansion of the charging infrastructure in public (and semi-public) spaces, and therefore constitutes the fifth minimum requirement. Also, the availability of parking data for combined parking and charging services ^72^ (‘Park and Charge’, P&C) is of high importance for interoperability and constitutes the sixth minimum requirement. Currently, there are no uniform standards for a uniform data exchange between stakeholders ^73^ (particularly for off-street) and, instead, multiple proprietary solutions are being used. A way to overcome this is to use open standard systems, e.g., Alliance for Parking Data Standards (APDS) ^74^, and to integrate P&C data into roaming platforms from the planning on. The availability of routing services for roaming and charging platforms ^75^ that allow EV driver an easy access to P&C services constitutes the seventh minimum requirement. Thus, open charging protocols should be used as well when providing routing services.

Closely related to all beforementioned requirements is the aspect of the cybersecurity of the charging points (as of every other product connected to the internet) ^76^. Cybersecurity is, in this sense, essential to the charging station itself, to CPOs and to the energy Distribution System Operators (DSOs) and constitutes the eighth minimum requirement. Some of the risks in terms of cybersecurity are “ *identity theft, data alteration, unauthorized access privileges, malware insertion, private & sensitive information theft, electricity flow manipulation and changes in operating parameters that may compromise charging stations’ safety”* ^77^. Security measures need, therefore, to be implemented for “ *communication, mobile apps, firmware updates and physical access points*” ^78^. To achieve the cybersecurity of the charging infrastructure, the implementation of unified protocols is essential, too. Moreover, the compliance with the data protection regulations ^79^ is of high importance ^80^.

As already mentioned in Section 3.1 about the global layer, communication and organisation is a key element for electromobility charging infrastructure. In the USER-CHI project, different products shall be developed and implemented in different demonstration sides. For example, INCAR, an interoperability, charging and parking platform, aims to ensure a barrier-free and operator-independent access for end-users, maximising EVSE availability, improving user convenience and providing roaming services along the two TEN-T corridors with transparent and flexible payment features. CLICK (Charging Location and Holistic Planning Kit) is another product developed within the USER-CHI project, that will be an easy-to-use question-and-answer online tool for the top-down location planning for charging infrastructure, which´s purpose is to optimise the location and planning of new charging infrastructure in cities. Therefore, CLICK will use data – like historic data, city data – to analyse locations for expanding electromobility charging hubs. Both products of USER-CHI are good examples of a first steps to meet the semantic requirements. The lessons-learned from the development of these products at the end of the project in 2024 will probably be a good adding to the requirements of INFRA and to what is needed to achieve interoperability in semantic layer.

**Figure 5.**
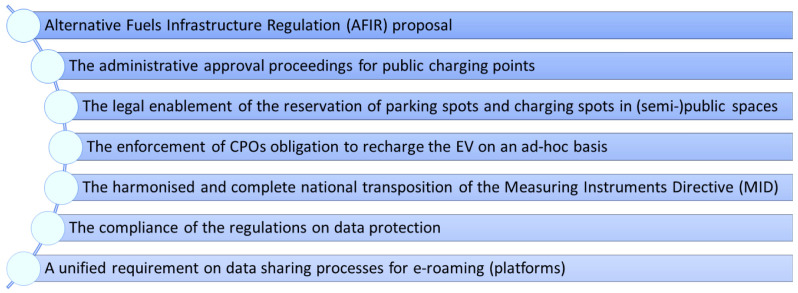
Summarised minimum requirements of the legal layer on basis of INFRA (Interoperability Framework). Own presentation. CPO, Charging Point Operators; EV, Electric Vehicle.

***3.2.3 Legal layer.*** The legal framework is meant to establish obligations for stakeholders (service providers, EV builders, governments, etc.), unify technical standards and set mechanisms to make charging easy and attractive for EV drivers. Therefore, an overall uniformity of the legal aspects for the deployment of a cross border charging infrastructure for EV is essential. In a first step, the guidelines and recommendations made in the INFRA deliverable within the legal layer were examined and summarized into minimum requirements. In this sense, a set of eight legal minimum requirements to achieve interoperability was consolidated as baseline for this paper and systematised as follows (see Figure 5). In a second step, further desk research was made in relation to the EU and national relevant laws and regulations to achieve the interoperability of a cross borders charging infrastructure as presented in INFRA. The desk research on the applicable legislation was expanded to any relevant legal aspect that could condition interoperability in other ways not considered in INFRA (for further information on the authorship see footnotes and citations).

As first legal requirement for interoperability, a harmonised national transposition of the AFID is necessary ^81^. The AFID regulates the roll out of the charging infrastructure for EVs and alternative fuels across the EU ^82^. Until now a harmonised transposition of the AFID into the national legislations of the EU state members has not been achieved. This and the fact that the AFID doesn´t include determinations for roaming for EV charging and charging protocols, constitute huge barriers for interoperability ^83^.

As consumers demand for EV is necessarily linked to the deployment of an interoperable charging infrastructure across the EU, the European Commission proposed a revision of the AFID that was included in the “Fit for 55” Package ^84^ published in July 2021. The AFID is planned to be repealed and replaced by the Alternative Fuels Infrastructure Regulation (AFIR), which will be directly applicable in the member States. This way, a uniform implementation of the obligations established at EU level in each member State is ensured. The AFIR proposal is also meant to fill some of the current legal gaps that hinder interoperability. In this sense, the actual adoption of this regulation would, so to say, guarantees the fulfilment of this first legal minimum requirement. The uptake of the AFIR will, in this way, introduce some changes and have impacts on different aspects of the other layers that condition interoperability ^85^.

Complementary to this is the inclusion of interoperability requirements in the national legal frameworks of the EU member states. In general, the national legislations of EU member states do not establish minimum requirements to ensure the interoperability of EV charging points operated by different CPOs ^86^. This requirement can´t be considered separately from the preceding requirement. Since the interoperability of the EV charging infrastructure across the EU depends completely on the uptake of minimum requirements at EU level (within all layers), the uncoordinated adoption of national requirements is rather detrimental. Thus, the actual adoption of the AFIR proposal, would guarantee a unified inclusion of the foreseen interoperability requirements in the national legal frameworks ^87^.

A second requirement, also considered to be essential to achieve interoperability in this sector, is for the administrative approval proceedings for public charging points to be optimised. Often the approval of new charging points in public spaces is competence of the municipalities and each municipality may, to a certain point, establish its own proceedings. These proceedings can be very tedious for all parties involved. In this regard, municipalities should reduce the complexity of their own authorisation proceedings ^88^.

The legal enablement of the reservation of parking spots and charging points in (semi-) public spaces is the third minimum legal requirement for interoperability ^89^. However, a uniform regulation of this matter at EU level has not been achieved yet. The AFIR proposal does not contain any provisions in this regard either. Therefore, a first step would be to conduct feasibility studies of this matter mainly at municipal level. An example of the implementation of P&C in an EU city is the case of Barcelona. The municipality of Barcelona developed a public service of parking spots on public roads for charging EV called “Endolla” ^90^. While using this service, users can make a reservation with 20 minutes in advance to charge their EV for up to 12 hours. In Berlin, and within the MEISTER project ^91^, four pilot P&C points have been installed with booking possibilities through the MEISTER Neighbourhood app ^92^. A similar service will be provided in the USER-CHI project with the beforementioned INCAR platform ^93^, where a reservation of P&C is possible ^94^.

The fourth minimum requirement is the enforcement of the CPOs obligation to offer the possibility to recharge the EV on an ad-hoc basis ^95^. This obligation is already foreseen in the AFID (Art. 4 Nr. 9) without further explanations on what *“on ad-hoc basis*” means or which services must be guaranteed. By contrast, it is established in the AFIR proposal that charging “ *on ad-hoc basis*” refers to “ *a recharging service purchased by an end user without the need for that end user to register, conclude a written agreement, or enter into a longer-lasting commercial relationship with the operator of that recharging point beyond the mere purchase of the service*” ^96^. To this respect, the AFIR proposal establishes that this service will have to be provided by all CPOs of public accessible charging points and, at the same time, that CPOs must guarantee the possibility for users to pay with credit card ^97^ at newly established charging points ^98^.

A fifth minimum requirement ^99^ is the harmonised and complete national transposition of the Measuring Instruments Directive (MID) ^100^ in all EU member states, or put differently, the enforcement of a directly binding legislation on metering devices in the EU. The AFID already established in Art. 4 Nr. 7 that, if technically feasible and economically reasonable, the recharging points accessible to the public shall “ *make use of intelligent metering systems”* in compliance with the Directive 2012/27/EU ^101^. Nevertheless, until now, there is no legal overall obligation for CPOs to use intelligent metering systems in publicly accessible charging points.

In this regard, the AFIR proposal highlights the importance of smart recharging points, which need to have specific hardware and software features that are necessary for the data exchange between stakeholders and end users ^102^, in combination with smart metering systems. This would have great benefits for users and would mean an easier integration of EV in the electric system. Thereafter, the AFIR proposal mentions that Member States should encourage, were technically and economically feasible, “ *the use of smart metering system for the recharging of EV at publicly accessible recharging stations*” ^103^. It also states that smart metering systems are essential to guarantee the availability of real-time data. This kind of data is needed “ *to ensure the stability of the grid and to encourage rational use of recharging services*” ^104^ as well as to ensure the accuracy of the billing information.

To the day, the requirements for smart metering devices are regulated by the Directive (EU) 2019/444 ^105^, which Member States should have already transposed into national law. In any case, if used, smart metering systems must comply with the requirements established in Art. 20 of the same Directive (EU) 2019/444. However, and even though smart metering systems could promote smart charging ^106^, the AFIR proposal does not lay down specific obligations regarding the use of smart metering systems within the EV charging infrastructure (unless it is a feature that smart charging points need to have, which is not established in the AFIR either) ^107^.

The compliance with the regulations on data protection regarding the personal data generated when EV drivers use public charging points ^108^ is the sixth minimum requirement. In this sense, the EU General Data Protection Regulation (GDPR) ^109^, which is directly applicable in all EU member states, and further nationally or regionally established obligations must be observed ^110^ (e.g., in German ‘ *Bundesdatenschutzgesetz*’ ^111^ or in Spanish ‘ *Ley de Protección de Datos’* ^112^). In this sense, some communication protocols like OCPP are configurable to comply with the GDPR provisions. However, the compliance with the GDPR is responsibility of the implementers of the OCPP ^113^.

In addition, the requirement of having binding obligations on data sharing processes between providers has been identified in the INFRA deliverable ^114^. The goal is to achieve interoperable systems (for all transactions that are necessary in the charging process) ^115^ that are not depending on a single provider. This is currently not possible because there are no legal obligations in this regard and stakeholders use proprietary systems that are mostly partly interoperable ^116^.

The importance of the data exchange between stakeholders is also recognised in the AFIR proposal ^117^. In this sense, the AFIR proposal mentions the importance of digitally connected and smart recharging points to achieve an interoperable charging infrastructure. These smart charging points need to have specific hardware and software features for the data exchange between stakeholders and end users ^118^. The specifications on these hardware and software features should be included in the legislation at EU level. Smart recharging is thus defined in Art. 2 Nr. 59 AFIR proposal as “ *a recharging operation in which the intensity of electricity delivered to the battery is adjusted in real-time, based on information received through electronic communication*” ^119^.

A digitally connected recharging point is, as established in Art. 2 Nr. 14 AFIR proposal, a charging point that “ *can send and receive information in real time, communicate bi-directionally with the electricity grid and the electric vehicle, and that can be remotely monitored and controlled, including to start and stop the recharging session and to measure electricity flows*”. Even though the AFIR proposal this way includes first references to the need for stakeholders to be able to exchange data, it does not establish a specific obligation for stakeholders to, for example, use open communication protocols, which are needed for smart charging ^120^ and that would ensure interoperability.

There is also a gap in relation to unified requirements on data sharing processes for e-roaming ^121^ or e-roaming platforms ^122^ for EV charging (seventh requirement) ^123^. The before mentioned overall situation in relation to data exchange between stakeholders across the EU foreseen in the AFIR proposal is the same for data sharing processes for roaming and roaming platforms.

On the other side, the existence of a uniform regulation of vehicle to grid charging/reverse charging is necessary (eighth requirement). There are currently non-binding international standards on this matter (ISO 15118-1:2019 ^124^ and IEC 60870-5-104 ^125^) and some countries (e.g., Germany and Spain) have started to act. As there are no obligations in force on this matter at EU level, a unified legislation on this matter is essential. ^126^

In this sense, the integration of this issue into the AFIR proposal is an important milestone. The AFIR proposal acknowledges in its Preamble (21) the importance of “ *bi-directional charging*” ^127^, to facilitate the integration of EV charging in the electric system. As already mentioned, the possibility of bi-directional charging, is a feature that digitally connecting charging points (Art. 2 Nr. 14 AFIR proposal) and, as set out in Art. 5 Nr. 7 AFIR proposal and if the AFIR proposal is finally adopted, CPOs will need to ensure the digital connectivity of charging points ^128^.

Related to these last aspects is the importance of the cybersecurity of EV charging points (as already mentioned in the semantic layer). However, there is still no unified cybersecurity protocol or regulation put in place. Therefore, there could be an overall lack of coordination between all stakeholders ^129^ (which is highly important for interoperability, as already mentioned in the global layer ^130^). Some countries, like the United Kingdom (UK), are already acting in this respect (even though not in the EU, it is still worth mentioning). In this sense, for example the UK plans to release new legislations on EV smart charging, that include cybersecurity requirements. The British Standard Institution already released two publicly available specifications (PAS) 1878 and 1879, that include security requirements that contribute to securing smart and V2G (Vehicle to Grid) charging infrastructure ^131^.

The use of encrypted communication, that no one can interfere with and/or alter, is a possible answer to this problem. The use of “smart contracts” ^132^ could pose a solution for the cybersecurity problems and might, also, simplify the contractual situation between service providers. There are multiple possible applications for smart contracts in blockchain-based systems ^133^. A special significance has the blockchain technology when it comes to the verifiability of transactions ^134^. Smart contracts could be used, in this sense, between stakeholders (Business to Business, B2B) to improve transparency, security, and effectivity ^135, 136^.

The use of smart legal contracts ^137^ to regularise relations between stakeholders and end consumers is also possible but it has some important disadvantages for the consumer due to their immutability ^138^. In this sense, the GDPR recognises the right to rectification (Art. 16 GDPR) and the right to erasure (‘ *right to be forgotten’,* Art. 17 GDPR) ^139^ and these rights are very difficult to guarantee with the use of smart contracts ^140^. In any case, there is no harmonised regulation at EU level on blockchain technology nor “smart contracts” and, with it, the interoperability of the charging infrastructure is not guaranteed with the use of these technologies either ^141^.

On the other hand, the incorporation into the EU legislation of time-of-use tariffs and, eventually, dynamic prices for EV charging are also aspects that would foster the roll out and scale up of electromobility ^142^. By offering price signals to users, it would be possible to shift the charging of EV to off-peak periods and optimise the use of the already available electricity without having to invest excessively in the expansion of the electricity production or distribution systems ^143, 144^.

## Conclusions

In conclusion, the interoperability of the charging infrastructure for EV across the EU can only be guaranteed if all member states integrate the described minimum requirements (from the global and the specific layers) in their legal framework and all involved stakeholders across the EU comply with them. For this to happen in a uniform way (also in terms of timing), the establishment of these minimum requirements needs to be pursued at the highest levels of the EU decision making instances.

Thereafter, the interoperability requirements of the specific layers (technical and semantic) need to be included in the EU legal framework. Interoperability requires, for example, that the necessary data for a better use of EVs is generated and exchanged uniformly. To ensure a convenient use of EV (enabled by the complete interoperability of the charging infrastructure) the accessibility issues (e.g., of data, hard- and software) need to be addressed uniformly by all member states of the EU. If these requirements are not implemented, achieving interoperability of the EV charging infrastructure in the EU (apart from those countries that are included in European projects) is going to be a challenge. Additionally, and because of the continuous technological developments, regulation must have the right scope to allow innovations, e.g., new technology solutions but as well-set relevant standards. Within the legal layer, there are currently two main aspects that are hindering the interoperability of the charging infrastructure for EV across the EU. The first is that there are still some important gaps in the EU legislation on the charging infrastructure of EV. Thus, some aspects like the use of a specific or interoperable data sharing protocol, bi-directional charging, smart charging points or the cybersecurity of the charging pints, consumer devices or grid systems have not found their way into the EU´s legislation yet (some of the might when and if the AFIR is enacted). The second is that, even though some aspects have already been included in the EU legislation, they have not been adequately transposed into the national legal frameworks of some of the member states. This causes wide disparities between countries. In this sense, the AFIR proposal is a step forward to overcome the current obstacles hindering the achievement of interoperability since no transposition into the national legal systems will be necessary and it is directly applicable. However, the AFIR proposal falls short in relation to some aspects, e.g., regarding the missing obligations in relation to interoperable data sharing protocols.

## 5 Outlook

Alongside all before mentioned aspects, EV users need to be considered more widely. Their convenience when charging and their acceptance of new technologies and regulations is essential for the needed uptake of EV. If the regulations and technical solutions (like uniform payment system) are not accepted by the EV users, interoperability will merely exist on paper. The success of the massive uptake of EV, strictly depends on solutions that satisfy EV user’s needs. The social aspects of the acceptance and uptake of electromobility are not mentioned in this paper but could be further analysed in a “social layer”. The following questions should be addressed there: “For which users is the infrastructure in EU interoperable and who can and who can’t use it?”, “what kind of barriers exist and what are the problems and reasons for overregulation or regulation gaps?”, “which and whose needs were integrated in the processes of developing a uniform charging infrastructure for EV?”

The prospective experiences within the USER-CHI project evaluation processes will help to answer some questions about EV users’ acceptance and could be linked to interoperability aspects in additional research. This research will most likely help to improve the interoperability of the EV charging infrastructure across the EU.

## Data availability

This study involves a literature review which was used to update and to complement the results of the existing D3.2 Interoperability Framework (INFRA) report of the user centric charging infrastructure (USER-CHI) project. The results of the D3.2 report have been published and are available on CORDIS: https://cordis.europa.eu/project/id/875187/results.

## Ethics and consent

Ethical approval and consent were not required.

## Footnotes

^1^ *International Energy Agency (IEA) (2021)*, Global EV Outlook 2021, online available at: https://www.iea.org/reports/global-ev-outlook-2021 (last consulted: 01.12.2021).

^2^ *International Energy Agency (IEA) (2021)*, Global EV Outlook 2021, online available at: https://www.iea.org/reports/global-ev-outlook-2021 (last consulted: 01.12.2021).

^3^ European Alternative Fuels Observatory (EAFO) (2022), Vehicles and fleets – AF (Alternative Fuels) Market share of new registrations, online available at: https://alternative-fuels-observatory.ec.europa.eu/transport-mode/road/european-union-eu27/vehicles-and-fleet (last consulted 20.04.2022).

^4^ *Wappelhorst, S./ Hall, D./ Nicholas, M./ Lutsey, N. (2020)*, Analysing Policies to Grow the Electric Vehicle Market in European Cities, White Paper, International Council on Clean Transportation, 2020, p. 3 f, online available at: https://theicct.org/wp-content/uploads/2021/06/EV_city_policies_white_paper_fv_20200224.pdf (last consulted: 13.04.2022).

^5^ Innovative solutions for USER centric CHarging Infrastructure. Grant agreement No 875187.

^6^ *Nowack, F./ Schmidt, J./ Freiberger, A./ Knoll, F. (2021)*, Interoperability Framework Implementation (INFRA). Deliverable (D3.2) of USER-CHI project (European Union's Horizon 2020 research and innovation programme under grant agreement No 875187), online available at: https://cordis.europa.eu/project/id/875187/results.

^7^ *Nowack, F./ Schmidt, J./ Freiberger, A./ Knoll, F. (2021), Interoperability Framework Implementation (INFRA). Deliverable (D3.2) of USER-CHI project (European Union's Horizon 2020 research and innovation programme under grant agreement No 875187), online available at: https://cordis.europa.eu/project/id/875187/results.*

^8^ Nowack, F./ Schmidt, J./ Freiberger, A./ Knoll, F. (2021), Interoperability Framework Implementation (INFRA). Deliverable (D3.2) of USER-CHI project (European Union's Horizon 2020 research and innovation programme under grant agreement No 875187), online available at: https://cordis.europa.eu/project/id/875187/results.

^9^ The most relevant individual requirements of all specific layers, in Nowack *et al* (2021), p. 15 ff.

^10^ Workshops held between December 14 ^th^ - 18 ^th^ 2020, in Nowack, F./ Schmidt, J./ Freiberger, A./ Knoll, F. (2021), Interoperability Framework Implementation (INFRA). Deliverable (D3.2) of USER-CHI project (European Union's Horizon 2020 research and innovation programme under grant agreement No 875187), online available at: https://cordis.europa.eu/project/id/875187/results, p. 16.

^11^ Workshops held between March 15 ^th^ - 19 ^th^ 2021, in Nowack, F./ Schmidt, J./ Freiberger, A./ Knoll, F. (2021), Interoperability Framework Implementation (INFRA). Deliverable (D3.2) of USER-CHI project (European Union's Horizon 2020 research and innovation programme under grant agreement No 875187), online available at: https://cordis.europa.eu/project/id/875187/results, p. 17.

^12^ The workshop data can be found in Nowack, F./ Schmidt, J./ Freiberger, A./ Knoll, F. (2021), Interoperability Framework Implementation (INFRA). Deliverable (D3.2) of USER-CHI project (European Union's Horizon 2020 research and innovation programme under grant agreement No 875187), online available at: https://cordis.europa.eu/project/id/875187/results, Annex 5, p. 106 ff.

^13^ In this paper the mentioned role schemes, guidelines and recommendations of the INFRA deliverable are summarised as minimum requirements.

^14^ *Naderifar, M./ Goli, H./Ghaljaei, F.* (2017), Snowball Sampling: A Purposeful Method of Sampling in Qualitative Research, Strides in Development of Medical Education, September 2017, S. 1-4, online available at: http://sdme.kmu.ac.ir/article_90598_3632edfb2e97c38d73c0bdea8753195c.pdf (last consulted: 13.04.2022).

^15^ *Greenhalgh, T.; Peacock, R.* (2005), Effectiveness and efficiency of search methods in systematic reviews of complex evidence: audit of primary sources, BMJ, November 2005, 1064-1065, online available at: https://www.bmj.com/content/bmj/331/7524/1064.full.pdf (last consulted: 13.04.2022)

^16^ *Kohli, A.* (2020), “Snowballing” in Systematic Literature Review, LinkedIn, July 2020, online available at: https://www.linkedin.com/pulse/snowballing-systematic-literature-review-amanpreet-kohli?articleId=6685292355240660992 (last consulted: 28.03.2022).

^17^ Communication from the Commission to the European Parliament, the Council, the European Economic and Social Committee and the Committee of the Regions 'Fit For 55': Delivering the EU's 2030 Climate Target on the way to climate neutrality (COM/2021/550 final), online available at: https://eur-lex.europa.eu/legal-content/EN/TXT/PDF/?uri=CELEX:52021DC0550&from=EN.

^18^ Nowack, F./ Schmidt, J./ Freiberger, A./ Knoll, F. (2021), Interoperability Framework Implementation (INFRA). Deliverable (D3.2) of USER-CHI project (European Union's Horizon 2020 research and innovation programme under grant agreement No 875187), online available at: https://cordis.europa.eu/project/id/875187/results, p. 20 ff.

^19^ Further to the developed overall role scheme and the specific role schemes for each demonstration city in Nowack, F./ Schmidt, J./ Freiberger, A./ Knoll, F. (2021), Interoperability Framework Implementation (INFRA). Deliverable (D3.2) of USER-CHI project (European Union's Horizon 2020 research and innovation programme under grant agreement No 875187), online available at: https://cordis.europa.eu/project/id/875187/results, p. 20–29.

^20^ The first two paragraphs are a summary of the INFRA deliverable, in Nowack, F./ Schmidt, J./ Freiberger, A./ Knoll, F. (2021), Interoperability Framework Implementation (INFRA). Deliverable (D3.2) of USER-CHI project (European Union's Horizon 2020 research and innovation programme under grant agreement No 875187), online available at: https://cordis.europa.eu/project/id/875187/results, p. 20 f.

^21^ The following paragraph shows the results of further desk research.

^22^ *NeMo* (2017), D1.1. Consolidated Version of the Use Cases and Actors’ Requirements, online available at: http://nemo-emobility.eu/wp-content/uploads/sites/10/2019/12/NeMo-D1.1-Use-Cases-and-Actors_Final.pdf (last consulted: 25.11.2021).

^23^ *Fanti, M.P./ Pedroncelli, G./ Roccotelli, M./ Mininel, S./ Stecco, G./ Ukovich, W.* (2018), Actors Interactions and Needs in the European Electromobility Network, online available at: https://arts.units.it/retrieve/handle/11368/2926616/231924/4461_11368_2926616_EUT.pdf (last consulted: 25.11.2021).

^24^ *Fanti, M.P./ Pedroncelli, G./ Roccotelli, M./ Mininel, S./ Stecco, G./ Ukovich, W.* (2018), Actors Interactions and Needs in the European Electromobility Network, online available at: https://arts.units.it/retrieve/handle/11368/2926616/231924/4461_11368_2926616_EUT.pdf (last consulted: 25.11.2021).

^25^ *Sadeghian, S/ Thébert, M./ Leurent, F./ Sajous, P.* (2012), Actors‘ positions and inclinations towards the electromobility system in France, p. 522, online available at: https://www.researchgate.net/publication/257716395_Actors%27_Positions_and_Inclinations_Toward_Electromobility_System_in_France (last consulted: 25.11.2021).

^26^The European Association for Electromobility, online available at: https://www.avere.org/.

^27^Electric Mobility Europe, online available at: https://www.electricmobilityeurope.eu/.

^28^European e-mobility Expertise Center, online available at: https://www.eemec.eu/.

^29^International Energy Agency, online available at: https://www.iea.org/.

^30^ European Alternative Fuels Observatory, online available at: https://eafo.eu/.

^31^ Nowack, F./ Schmidt, J./ Freiberger, A./ Knoll, F. (2021), Interoperability Framework Implementation (INFRA). Deliverable (D3.2) of USER-CHI project (European Union's Horizon 2020 research and innovation programme under grant agreement No 875187), online available at: https://cordis.europa.eu/project/id/875187/results , p. 37-41.

^32^ This paragraph summarises information of the INFRA deliverable; Nowack, F./ Schmidt, J./ Freiberger, A./ Knoll, F. (2021), Interoperability Framework Implementation (INFRA). Deliverable (D3.2) of USER-CHI project (European Union's Horizon 2020 research and innovation programme under grant agreement No 875187), online available at: https://cordis.europa.eu/project/id/875187/results, p. 48 f.

^33^ Nowack, F./ Schmidt, J./ Freiberger, A./ Knoll, F. (2021), Interoperability Framework Implementation (INFRA). Deliverable (D3.2) of USER-CHI project (European Union's Horizon 2020 research and innovation programme under grant agreement No 875187), online available at: https://cordis.europa.eu/project/id/875187/results, p. 39 f.

^34^ Nowack, F./ Schmidt, J./ Freiberger, A./ Knoll, F. (2021), Interoperability Framework Implementation (INFRA). Deliverable (D3.2) of USER-CHI project (European Union's Horizon 2020 research and innovation programme under grant agreement No 875187), online available at: https://cordis.europa.eu/project/id/875187/results, p. 29 f.

^35^ Directive 2014/94/EU of the European Parliament and of the Council of 22 October 2014 on the deployment of alternative fuels infrastructure. Online available at: https://eur-lex.europa.eu/legal-content/EN/TXT/PDF/?uri=CELEX:32014L0094&from=en.

^36^ The first three paragraphs of the technical layer are based on the input of the INFRA deliverable. The link to the Alternative Fuels Infrastructure Directive (AFID) is the author’s own adding.

^37^ Light electric vehicles, like e-bikes or e-scooters.

^38^ Nowack, F./ Schmidt, J./ Freiberger, A./ Knoll, F. (2021), Interoperability Framework Implementation (INFRA). Deliverable (D3.2) of USER-CHI project (European Union's Horizon 2020 research and innovation programme under grant agreement No 875187), online available at: https://cordis.europa.eu/project/id/875187/results, p. 40 f.

^39^ *Singh, K./ Mishra, A./ Singh, B./ Sahay, K.* (2021), Solar powered battery charging scheme for light electric vehicles (LEVs), International Journal of Emerging Electric Power Systems 2021, p. 101-111, https://doi.org/10.1515/ijeeps-2020-0200.

^40^ *Sharma, U./ Singh, B*. (2021), Seamless Integration of An Onboard Charger in a Smart Home, 2021 IEEE 4th International Conference on Computing, Power and Communication Technologies (GUCON) 2021, p. 1-6. , doi: 10.1109/GUCON50781.2021.9573859.

^41^ *“SEVs according to EU regulation No. 168/2013 as well as electric cargo bikes. Cargo bikes can be two-, three- or four-wheeled with and without insurance tags“,* in *Eisenmann, C. / Gruber, J./ Brost, M./ Ewert, A./ Stieler, S./ Gicklhorn, K. in: Ewert A./ Schmid S./ Brost M./ Davies H./ Vinckx L*., Small Electric Vehicles, 2021, p. 128.

^42^ This paragraph and the following paragraphs of the technical layer reflect the authors input resulting from the desk research.

^43^ *Inter alia Panchal, C/; Stegen, S./ Lu, J.* (2018), Review of static and dynamic wireless electric vehicle charging system, Engineering Science and Technology, an International Journal, 2018, p. 922-937, online available at: https://reader.elsevier.com/reader/sd/pii/S221509861830154X?token=DBE7E99C6D012B0D520F0051374960BA828EC954AC108FB0D03A672C4E39F5DA176E359815B3BA0D7B94B229D4940C2E&originRegion=eu-west-1&originCreation=20220413121037 (last consulted: 13.04.2022), *Fett, D./ Ensslen, A./ Jochem, P./ Fichtner, W.* (2018), A Survey on User Acceptance of Wireless Electric Vehicle Charging, World Electric Vehicle Journal 9, 36, August 2018, p. 1-12, online available at: https://mdpi-res.com/d_attachment/wevj/wevj-09-00036/article_deploy/wevj-09-00036-v2.pdf (last consulted: 13.04.2022). On this matter also *Shi, B./ Yang, F./ Wei, B./ Ouyang, M.* (2021), Electrical Interoperability Evaluating of Wireless Electric Vehicle Charging Systems Based on Impedance Space, World Electric Vehicle Journal, 12, 245, November 2021, p. 1-14, online available at: https://mdpi-res.com/d_attachment/wevj/wevj-12-00245/article_deploy/wevj-12-00245.pdf (last consulted: 13.04.2022).

^44^ *Vidhya, K./ Sharmeela, C./ Balaji, S./ Elango, S./ Sanjeevikumar, P./ Bhaskar, M.S.* (2021), Techno-Economic Aspects of the Wireless EV Charging System, IECON 2021 – 47th Annual Conference of the IEEE Industrial Electronics Society 2021, p. 1-6.

^45^ *Gadge, C./ Khule, Dr. S. S.* (2021), E-Vehicle Charging Infrastructure Based on Inductive Wireless Power Transfer Scheme, International Journal of Research in Electronics and Computer Engineering, Vol. 9, Jan-March 2021, p. 74-77.

^46^ *Pompigna, A./ Mauro, R.* (2021), Smart roads: A state of the art of highways innovations in the Smart Age, Engineering Science and Technology, an international Journal, 25, 2022, p. 1-15, online available at: https://reader.elsevier.com/reader/sd/pii/S2215098621000872?token=31CD6784C811F33C48E5244938B9D146599456A693CE5E76A7A2699ACEF93F84E4579A96D07277DBFFE796B79474D3C0&originRegion=eu-west-1&originCreation=20220413120616 (last consulted: 13.04.2022).

^47^ Inter alia *Calearo, L./ Ziras, H./ Sevdari, K./ Marinelli, M.* (2021), Comparison of Smart Charging and Battery Energy Storage System for a PV Prosumer with an EV, 2021 IEEE PES Innovative Smart Grid Technologies Europe (ISGT Europe) 2021, p. 1-6. Also on this matter *Shahzad, U.* (2022), Smart Grid and Electric Vehicle: Overview and Case Study, Journal of Electrical Engineering, Electronic, Control and Computer Science – JEEECCCS, Vol. 8, 2022, p. 1-6, online available at: https://www.researchgate.net/profile/Umair-Shahzad/publication/353667007_Smart_Grid_and_Electric_Vehicle_Overview_and_Case_Study/links/610968080c2bfa282a201746/Smart-Grid-and-Electric-Vehicle-Overview-and-Case-Study.pdf?origin=publication_detail (last consulted 13.04.2022).

^48^ See section 4.3.2 *Legal Layer*.

^49^ *Gadgune, S. Y./ Hewalekar, S. N.* (2020), Progression of Smart Metering Infrastructure for Electric Vehicle Charging Station, 2020 Third International Conference on Smart Systems and Inventive Technology (ICSSIT) 2020, p. 370-375.

^50^ *Cavalieri, S.* (2021), Semantic Interoperability between IEC 61850 and oneM2M for IoT-Enabled Smart Grids, Sensors 2021, 21, 2571, p. 1-28, online available at: https://www.ncbi.nlm.nih.gov/pmc/articles/PMC8038825/pdf/sensors-21-02571.pdf (last consulted: 25.11.2021).

^51^ Nowack *Nowack, F./ Schmidt, J./ Freiberger, A./ Knoll, F. (2021), Interoperability Framework Implementation (INFRA). Deliverable (D3.2) of USER-CHI project (European Union's Horizon 2020 research and innovation programme under grant agreement No 875187), online available at: https://cordis.europa.eu/project/id/875187/results*, p. 29-37.

^52^ Standardized through IEC 63119.

^53^ Standardized through IEC 63110.

^54^ Nowack, F./ Schmidt, J./ Freiberger, A./ Knoll, F. (2021), Interoperability Framework Implementation (INFRA). Deliverable (D3.2) of USER-CHI project (European Union's Horizon 2020 research and innovation programme under grant agreement No 875187), online available at: https://cordis.europa.eu/project/id/875187/results, p. 29 f.

^55^ Nowack, F./ Schmidt, J./ Freiberger, A./ Knoll, F. (2021), Interoperability Framework Implementation (INFRA). Deliverable (D3.2) of USER-CHI project (European Union's Horizon 2020 research and innovation programme under grant agreement No 875187), online available at: https://cordis.europa.eu/project/id/875187/results, p. 30 f.

^56^ Nowack, F./ Schmidt, J./ Freiberger, A./ Knoll, F. (2021), Interoperability Framework Implementation (INFRA). Deliverable (D3.2) of USER-CHI project (European Union's Horizon 2020 research and innovation programme under grant agreement No 875187), online available at: https://cordis.europa.eu/project/id/875187/results, p. 31 f.

^57^ Nowack, F./ Schmidt, J./ Freiberger, A./ Knoll, F. (2021), Interoperability Framework Implementation (INFRA). Deliverable (D3.2) of USER-CHI project (European Union's Horizon 2020 research and innovation programme under grant agreement No 875187), online available at: https://cordis.europa.eu/project/id/875187/results) , p. 33.

^58^ The two first paragraphs summarise the results of the INFRA deliverable.

^59^ Further on blockchain in the section 4.3.2 *Legal layer*.

^60^ Ladesäulenverordnung vom 9. März 2016 (BGBl. I S. 457), die zuletzt durch Artikel 2 der Verordnung vom 2. November 2021 (BGBl. I S. 4788) geändert worden ist.

^61^ *Röckle, F./ Schulz,T*. (2021), Leveraging User Preferences to Develop Profitable Business Models for Electric Vehicle Charging, World Electric Vehicle Journal 2021, 12, 60, p. 1-16, online available at: https://mdpi-res.com/d_attachment/wevj/wevj-12-00060/article_deploy/wevj-12-00060.pdf (last consulted: 13.04.2022).

^62^ *Initiative deutscher Zahlungssysteme (IDZ)* (2021), Survey on e-mobility among consumers - Card payment needs to be possible at charging stations throughout Europe, from 30.11.2021, online available at: https://www.presseportal.de/en/pm/127450/5086776 (last consulted: 01.12.2021).

^63^ This paragraph reflects the results of further desk research.

^64^ See also Alternative Fuels Infrastructure Directive (AFID) in section 4.2.3. *Legal layer*.

^65^ European Commission, Proposal for a Regulation of the European Parliament and of the Council on the deployment of alternative fuels infrastructure and repealing Directive 2014/94/EU of the European Parliament and of the Council (COM/2021/559 final), online available at: https://eur-lex.europa.eu/legal-content/EN/TXT/?uri=CELEX%3A52021PC0559 (last consulted: 02.12.2021).

^66^ *Initiative deutscher Zahlungssysteme (IDZ)* (2021), Survey on e-mobility among consumers - Card payment needs to be possible at charging stations throughout Europe, from 30.11.2021, online available at: https://www.presseportal.de/en/pm/127450/5086776 (last consulted: 01.12.2021).

^67^ *Transport & Environment* (2021), AFIR: providing infrastructure to make transport fit for 55, Briefing, November 2021, p. 2, online available at: https://www.transportenvironment.org/wp-content/uploads/2021/11/20211004_AFIR_Briefing.pdf (last consulted: 01.12.2021).

^68^ *The European Automobile Manufacturers’ Association* (ACEA) (2021), ACEA Position Paper - Proposal for the Alternative Fuels Infrastructure Regulation (AFIR), November 2021, p. 14, online available at: https://www.acea.auto/files/ACEA_Position_Paper-Alternative_Fuels_Infrastructure_Regulation.pdf (last consulted: 02.12.2021).

^69^ This paragraph reflects the results of further desk research.

^70^ This paragraph summarises the results of the INFRA deliverable.

^71^ Nowack, F./ Schmidt, J./ Freiberger, A./ Knoll, F. (2021), Interoperability Framework Implementation (INFRA). Deliverable (D3.2) of USER-CHI project (European Union's Horizon 2020 research and innovation programme under grant agreement No 875187), online available at: https://cordis.europa.eu/project/id/875187/results , p. 33 f.

^72^ Nowack, F./ Schmidt, J./ Freiberger, A./ Knoll, F. (2021), Interoperability Framework Implementation (INFRA). Deliverable (D3.2) of USER-CHI project (European Union's Horizon 2020 research and innovation programme under grant agreement No 875187), online available at: https://cordis.europa.eu/project/id/875187/results , p. 33 f.

^73^ Specially for operators of parking facilities (especially off-street), in Nowack *et al* (2021), p. 34f.

^74^ The Alliance for Parking Data Standards (APDS) is an international organisation aiming at establishing a global standard for data elements related to providing various parking services.

^75^ Nowack, F./ Schmidt, J./ Freiberger, A./ Knoll, F. (2021), Interoperability Framework Implementation (INFRA). Deliverable (D3.2) of USER-CHI project (European Union's Horizon 2020 research and innovation programme under grant agreement No 875187), online available at: https://cordis.europa.eu/project/id/875187/results , p. 36 f.

^76^ The information contained in the following two paragraphs reflects the results from the authors own desk research.

^77^ DEKRA (2021), The importance of cybersecurity in electric vehicle charging stations, June 2021, online available at: https://www.dekra-product-safety.com/en/importance-cybersecurity-electric-vehicle-charging-stations.

^78^ DEKRA (2021), The importance of cybersecurity in electric vehicle charging stations, June 2021, online available at: https://www.dekra-product-safety.com/en/importance-cybersecurity-electric-vehicle-charging-stations.

^79^ European Commission, Data protection in the EU, online available at https://ec.europa.eu/info/law/law-topic/data-protection/data-protection-eu_en.

^80^ For more information on the data protection requirements see section 4.3.2 *Legal layer*.

^81^ Nowack, F./ Schmidt, J./ Freiberger, A./ Knoll, F. (2021), Interoperability Framework Implementation (INFRA). Deliverable (D3.2) of USER-CHI project (European Union's Horizon 2020 research and innovation programme under grant agreement No 875187), online available at: https://cordis.europa.eu/project/id/875187/results, p. 41f.

^82^ This paragraph is a result from the INFRA deliverable.

^83^ The result of a not harmonized transposition of the Alternative Fuels Infrastructure Directive (AFID) is a result of the desk research.

^84^ European Commission, Proposal for a Regulation of the European Parliament and of the Council on the deployment of alternative fuels infrastructure and repealing Directive 2014/94/EU of the European Parliament and of the Council (COM/2021/559 final).

^85^ This paragraph reflects the results of the authors desk research.

^86^ Nowack, F./ Schmidt, J./ Freiberger, A./ Knoll, F. (2021), Interoperability Framework Implementation (INFRA). Deliverable (D3.2) of USER-CHI project (European Union's Horizon 2020 research and innovation programme under grant agreement No 875187), online available at: https://cordis.europa.eu/project/id/875187/results, p. 42 f.

^87^ The rest of the paragraph is the authors own input on the subject.

^88^ Nowack, F./ Schmidt, J./ Freiberger, A./ Knoll, F. (2021), Interoperability Framework Implementation (INFRA). Deliverable (D3.2) of USER-CHI project (European Union's Horizon 2020 research and innovation programme under grant agreement No 875187), online available at: https://cordis.europa.eu/project/id/875187/results, p. 43 f.

^89^ Based on the input of the INFRA deliverable, Nowack *et al* (2021), p. 44 f.

^90^ Endolla Barcelona, Terms and conditions of use, Seventh condition, online available at: https://www.endolla.barcelona/es/condiciones-de-uso (last consulted: 01.12.2021).

^91^ MEISTER is also a Horizon 2020 research and innovation project (grant agreement No 769052). It aims at changing the paradigm in the electromobility market by providing to interoperable platforms and services for easy, convenient, and barrier-free access to charging, billing, and smart grid service, online available at: https://meisterproject.eu/ (last consulted: 13.04.2022).

^92^ Smart P&C pilot, MEISTER, online available at: https://meisterproject.eu/berlin-begins-piloting-smart-park-charge/ (last consulted: 13.04.2022).

^93^ See section 3.2.2.

^94^ The rest of the paragraph reflects the results of the authors desk research.

^95^ The paragraph is the result of the authors own desk research.

^96^ Art. 2 Nr. 40 Alternative Fuels Infrastructure Regulation (AFIR) proposal.

^97^ Art. 5 Nr. 2 Alternative Fuels Infrastructure Regulation (AFIR) proposal.

^98^ This date will be determined when the Alternative Fuels Infrastructure Regulation (AFIR) proposal is officially enacted.

^99^ The fifth requirement based on the INFRA deliverable. All further information on Measuring Instruments Directive (MID) and Alternative Fuels Infrastructure Regulation (AFIR) proposal in this paragraph and the following paragraph is a result of the authors own desk research.

^100^ Directive 2014/32/EU of the European Parliament and of the Council of 26 February 2014 on the harmonisation of the laws of the Member States relating to the making available on the market of measuring instruments (recast).

^101^ Directive 2012/27/EU of the European Parliament and of the Council of 25 October 2012 on energy efficiency, amending Directives 2009/125/EC and 2010/30/EU and repealing Directives 2004/8/EC and 2006/32/EC.

^102^ Alternative Fuels Infrastructure Regulation (AFIR) proposal, Preamble (19).

^103^ Alternative Fuels Infrastructure Regulation (AFIR) proposal, Preamble (20).

^104^ Alternative Fuels Infrastructure Regulation (AFIR) proposal, Preamble (20).

^105^ Directive (EU) 2019/944 of the European Parliament and of the Council of 5 June 2019 on common rules for the internal market for electricity and amending Directive 2012/27/EU.

^106^ *Eyres, R.* (2021), Smart charging: now is the time to plan, September 2021, online available at: https://www.engineerlive.com/content/smart-charging-now-time-plan (last consulted on 13.04.2021).

^107^ This paragraph reflects the results of the desk research.

^108^ Nowack, F./ Schmidt, J./ Freiberger, A./ Knoll, F. (2021), Interoperability Framework Implementation (INFRA). Deliverable (D3.2) of USER-CHI project (European Union's Horizon 2020 research and innovation programme under grant agreement No 875187), online available at: https://cordis.europa.eu/project/id/875187/results, p. 46 f.

^109^ Regulation (EU) 2016/679 of the European Parliament and of the Council of 27 April 2016 on the protection of natural persons with regard to the processing of personal data and on the free movement of such data, and repealing Directive 95/46/EC.

^110^ This statement is a result of the INFRA deliverable. The following phrases of this paragraph are results of the desk research.

^111^ Bundesdatenschutzgesetz vom 30. Juni 2017 (BGBl. I S. 2097), das durch Artikel 10 des Gesetzes vom 23. Juni 2021 (BGBl. I S. 1858) geändert worden ist.

^112^ Ley Orgánica 3/2018, de 5 de diciembre, de Protección de Datos Personales y garantía de los derechos digitales.

^113^ Open Charge Alliance, online available at: https://www.openchargealliance.org/about-us/ocpp-and-gdpr/ (last consulted: 13.04.2022).

^114^ Nowack, F./ Schmidt, J./ Freiberger, A./ Knoll, F. (2021), Interoperability Framework Implementation (INFRA). Deliverable (D3.2) of USER-CHI project (European Union's Horizon 2020 research and innovation programme under grant agreement No 875187), online available at: https://cordis.europa.eu/project/id/875187/results, p. 47 f.

^115^ For example, reservation, authentication, accounting, and billing, in Nowack *et al* (2021), p. 46.

^116^ Based on the input of the INFRA deliverable.

^117^ Alternative Fuels Infrastructure Regulation (AFIR) proposal, Preamble (19).

^118^ Alternative Fuels Infrastructure Regulation (AFIR) proposal, Preamble (19).

^119^ This paragraph and the next one about the Alternative Fuels Infrastructure Regulation (AFIR) are the result of the authors desk research.

^120^ Anisie, A./ Boshell, F./ Sesma, J. (2019), International Renewable Energy Agency (IRENA), Innovation landscape brief: Electric-vehicle smart charging, p. 16, online available at: https://irena.org/-/media/Files/IRENA/Agency/Publication/2019/Sep/IRENA_EV_smart_charging_2019.pdf?la=en&hash=E77FAB7422226D29931E8469698C709EFC13EDB2 (last consulted on 13.04.2021).

^121^ “e-roaming “refers to “ *the exchange of data and payments between the operator of a recharging or refuelling point and a mobility service provider from which an end user purchases a recharging service*“, Art. 2 Nr. 21 Alternative Fuels Infrastructure Regulation (AFIR) proposal.

^122^ “e-roaming platform” refers to “ *a platform connecting market actors, notably mobility service providers and operators of recharging or refuelling points, to enable services between them, including e-roaming*”, Art.2 Nr.22 Alternative Fuels Infrastructure Regulation (AFIR) proposal.

^123^ The idea of the seventh minimum requirement is based on the input of the INFRA deliverable, Nowack *et al* (2021), p. 48 f. The further development of this idea is input of the authors.

^124^ International Organization for Standardization, ISO 15118-1:2019, Road vehicles — Vehicle to grid communication interface, preview available at: https://www.iso.org/obp/ui/#iso:std:iso:15118:-1:ed-2:v1:en (last consulted: 13.04.2022) .

^125^International Electrotechnical Commission, IEC 60870-5-104, Transmission Protocols, preview online available at: https://webstore.iec.ch/preview/info_iec60870-5-104%7Bed2.0%7Den_d.pdf (last consulted: 13.04.2022).

^126^ Input from the INFRA deliverable, Nowack, F./ Schmidt, J./ Freiberger, A./ Knoll, F. (2021), Interoperability Framework Implementation (INFRA). Deliverable (D3.2) of USER-CHI project (European Union's Horizon 2020 research and innovation programme under grant agreement No 875187), online available at: https://cordis.europa.eu/project/id/875187/results, p. 49 f.

^127^ “bi-directional recharging” refers to “ *smart recharging operation where the direction of the electricity flow may be reversed, allowing that electricity flows from the battery to the recharging point it is connected to*”, Art. 2 Nr. 9 AFIR proposal.

^128^ This paragraph and the following constitute the authors own input.

^129^ *Lawrence, C.* (2021), What's holding back electric vehicle charging security?, Power&Beyond, April 2021, online available at: https://www.power-and-beyond.com/whats-holding-back-electric-vehicle-charging-security-a-1019375/ (last consulted: 15.12.2021).

^130^ See section 4.1.

^131^ *Metere, R./ Neaimeh, M./ Morisset, C./ Czekste, R.M./ Bellekens, X./ Maple, C.* (2021), Securing the Electric Vehicle Charging Infrastructure, State-of-the-art review and recommendations with a focus on smart charging and vehicle-to-grid, 2021, p. 1-39, online available at: https://www.researchgate.net/profile/Ricardo-Czekster/publication/351448785_Securing_the_Electric_Vehicle_Charging_Infrastructure/links/609b9a4692851c9eaadd1949/Securing-the-Electric-Vehicle-Charging-Infrastructure.pdf?origin=publication_detail (last consulted: 13.04.2022).

^132^ A “smart contract” describes a software that is programmed to, under specific conditions (measurable and/or documentable), control, keep record of or even generate legally relevant contractual relations, in *Schrey, J./ Thalhofer, T.* (2017), Rechtliche Aspekte der Blockchain, NJW 2017, p. 1431-1436. The contract information is defined in the programme code under the principle “code is law”, in *Schiller, K.* (2018), Was sind Smart Contracts? Definition und Erklärung, November 2018, online available at: https://blockchainwelt.de/smart-contracts-vertrag-blockchain/ (last consulted: 10.11.2021). The contractual obligations and the consequences of breaches of duty are programmed as "if-then rules": if a condition specified in the contract is fulfilled, this automatically leads to the previously determined consequence, *Kipker, D./ Birreck, P./ Niewöhner, M./ Schnorr, T.* (2020), Rechtliche und technische Rahmenbedingungen des „Smart contracts“, MMR - Zeitschrift für IT-Recht und Digitalisierung 2020, p. 509-513, online available at: https://www.nutrisafe.de/wp-content/uploads/2020/10/3-Rechtliche-und-technische-Rahmenbedingungen-der-%E2%80%9ESmart-Contracts%E2%80%9C.pdf (last consulted: 10.11.2021). Only if both parties comply (“pre-check”), then the contract is automatically concluded, in Nowack *et al* (2021), p. 51. Smart contracts could increase the efficiency and security of digital transactions, in Kipker *et al* (2020), p. 513.

^133^ Blockchain is a distributed database; it is a specific way of storing transaction or payment data. The data is written in a "block ", as soon as its capacity is reached, the data are written in the next block and so on. This block in turn refers to the previous one, so that a chain of blocks is created, the "blockchain", in Schrey *et al* (2017), p. 1431.

^134^ *Schrey, J./ Thalhofer, T.* (2017), Rechtliche Aspekte der Blockchain, NJW 2017, p. 1431-1436. p. 1431.

^135^ The advantages of “smart contracts” are that (1) they are executed automatically, when the required event occurs, (2) contract partners get real time information, and (3) they are very secure against forgery, as no central database can be attacked or manipulated, in *Schiller, K.* (2018).

^136^ The whole paragraph is based on input from the INFRA deliverable, in Nowack, F./ Schmidt, J./ Freiberger, A./ Knoll, F. (2021), Interoperability Framework Implementation (INFRA). Deliverable (D3.2) of USER-CHI project (European Union's Horizon 2020 research and innovation programme under grant agreement No 875187), online available at: https://cordis.europa.eu/project/id/875187/results, p. 50 ff.

^137^ The idea of smart (legal) contracts is a result of the INFRA deliverable. The input about the right to rectification and the right to erasure in the GDPR is a result of the desk research.

^138^ The immutability of smart contracts is, at the same time, an advantage and disadvantage.

^139^ Regulation (EU) 2016/679 of the European Parliament and of the Council of 27 April 2016 on the protection of natural persons with regard to the processing of personal data and on the free movement of such data, and repealing Directive 95/46/EC.

^140^ The information of this last sentence is input from the authors desk research.

^141^ Input from the INFRA deliverable.

^142^ Input not contemplated in INFRA.

^143^ International Renewable Energy Agency (IRENA)(2019), Innovation outlook: Smart charging for electric vehicles, p. 9, online available at: https://www.irena.org/-/media/Files/IRENA/Agency/Publication/2019/May/IRENA_Innovation_Outlook_EV_smart_charging_2019.pdf (last consulted: 13.04.2021).

^144^ Author‘s own input resulting from the desk research.

