# Supplementary material for: An Interoperability Framework for electromobility (INFRA): The main results from the USER-CHI framework implementation in a new spotlight
Source: Open Res Eur. 2022 May 25;2:65. [Version 1] doi: 10.12688/openreseurope.14510.1 (PMC10446027; doi:10.12688/openreseurope.14510.1)
Supplement: Peer review history

# Peer review: Reviewer response for version 1

**DOI:** 10.21956/openreseurope.15663.r30348

**Published:** 2022-10-26

**Authors:** Natascia Andrenacci

**Affiliations:** 1 Ente per le Nuove Tecnologie, l'Energia e l'Ambiente (ENEA), Rome, Italy

The issue of interoperability in the field of electric mobility is of primary interest to increase the adoption of electric vehicles. The article investigates the current barriers to the interoperability of charging points and indicates some actions to mitigate or overcome the problem. The article is well structured and sufficiently clear in the exposition.

- Generally, I would recommend limiting repeated references to deliverable 4.3.
- Regarding the management of footnotes: Being structured as a paragraph of bibliography/references, I would recommend inserting each source only once in the list, and citing it if necessary in the text.
- Since the acronyms have been defined in a specific table, it is not necessary to repeat the definition several times in the text (e.g., INFRA is reported also in full in many captions), limiting the explanation to the first citation.

Is the study design appropriate and does the work have academic merit?

Yes

Is the work clearly and accurately presented and does it cite the current literature?

Yes

If applicable, is the statistical analysis and its interpretation appropriate?

Not applicable

Are all the source data underlying the results available to ensure full reproducibility?

No source data required

Are the conclusions drawn adequately supported by the results?

Yes

Are sufficient details of methods and analysis provided to allow replication by others?

Yes

Reviewer Expertise:

Electric mobility; charging infrastructures; batteries; batteries ageing; data analysis

I confirm that I have read this submission and believe that I have an appropriate level of expertise to confirm that it is of an acceptable scientific standard.

# Peer review: Reviewer response for version 1

**DOI:** 10.21956/openreseurope.15663.r29474

**Published:** 2022-06-27

**Authors:** Dr. Helga Jonuschat

**Affiliations:** 1 Dornier Consulting International GmbH, Berlin, Germany

This report provides a thorough overview on the status quo of the challenges of achieving interoperability in the context of the charging infrastructure roll-out in Europe. It suggests a structured analysis along four layers (organisational, technical, semantic, legal) and provides insight on the USER-CHI project results for each layer. The study design is suitable for the research question and methods and sources have been presented thoroughly to ensure replicability. The paper is clearly structured and written, although references to project details (e.g. deliverables) are not important for the wider public. Overall, this paper addresses a complex topic and provides very useful insights on the status quo of interoperability of charging networks in the EU.

Is the study design appropriate and does the work have academic merit?

Yes

Is the work clearly and accurately presented and does it cite the current literature?

Yes

If applicable, is the statistical analysis and its interpretation appropriate?

Not applicable

Are all the source data underlying the results available to ensure full reproducibility?

No source data required

Are the conclusions drawn adequately supported by the results?

Yes

Are sufficient details of methods and analysis provided to allow replication by others?

Yes

Reviewer Expertise:

Electric mobility, interoperability, user requirements, charging infrastructure roll-out, policy analysis

I confirm that I have read this submission and believe that I have an appropriate level of expertise to confirm that it is of an acceptable scientific standard.
